# Editorial: Gene Regulation as a Driver of Adaptation and Speciation

**DOI:** 10.3389/fgene.2021.793933

**Published:** 2021-11-12

**Authors:** Deborah A. Triant, Katja Nowick, Ekaterina Shelest

**Affiliations:** ^1^ Department of Biochemistry & Molecular Genetics, University of Virginia, Charlottesville, VA, United States; ^2^ Institute for Biology, Freie Universität Berlin, Berlin, Germany; ^3^ Centre for Enzyme Innovation, University of Portsmouth, Portsmouth, United Kingdom

**Keywords:** gene regulatory factors, adaptation, speciation, transcription factors, biodiversity, evolution

One fundamental goal in evolutionary biology is to understand the interaction between adaptation and speciation and how it can generate and maintain biodiversity (Wolf et al., 2010). Speciation leads to the diversification of lineages, whereas adaptation maximizes the survival and reproductive success of organisms in an ever-changing environment, thereby further increasing diversification. Genetic approaches have been critical for examining the molecular basis of adaptation and speciation. The availability of an increasing number of genome assemblies allows for the identification of genomic components that underlie these processes, providing new insights into the proximate mechanisms of diversification and deeper understanding of biodiversity evolution.

Recent discoveries suggest that gene regulation plays an important role in speciation and adaptive diversification (Jones et al., 2012; Mack et al., 2018). We present this collection of papers, eight of original research, one opinion and one review, that explore genomic features that foster divergence in gene regulation and how evolutionary changes of regulation systems impact adaptation and speciation. The incorporation of gene regulation in evolutionary processes is multifaceted, and so are the approaches to its understanding. This multiplicity is reflected by the scope of the articles in our collection, ranging from the interplay between regulation and evolutionary patterns on a molecular level (e.g., Aviña-Padilla et al., Mohanty) to the investigation of adaptations of organisms to certain conditions (Wang et al.). Changes caused by epigenetics, structural variants, transcription factors (TFs) and regulatory sequences that elicit modifications in gene expression and drive adaptation and diversification are also explored. Here, we briefly summarize the authors’ findings.

We start with contributions that investigated genome architecture. In interspecific hybridization in *Saccharomyces* yeast described by Hovhannisyan et al., the coexistence of two distinct genomes after hybridization is supported by the limited regulatory interference on broad transcriptional changes. This may explain the unusually strong ability of these organisms to successfully hybridize. In instances of genome duplications, the genomic history can play a crucial role in adaptation. As shown by Zhao et al., genes can be regulated differently depending on whether or not they have been duplicated. The authors evaluated transcriptional changes in cultivated and wild *Glycine* soybeans with highly duplicated genomes after whole genome duplications. They found more *trans*-only regulation in duplicated genes than in singletons, especially in tandem duplications. Their results suggest that genes with different duplication modes accumulate different types of regulatory divergence.

Two papers show how changes on a chromosomal level can be instrumental in adaptation and speciation. Pabuayon et al. presented plant architectural modifications created through transgressive segregation in Pokkali rice. The authors examined morpho-developmental and physiological profiles and used transcriptomic data of recombinant inbred lines varying in salt tolerance to build genetic networks. The network analysis indicated novel adaptive architectures correlating with levels of salinity stress. The study demonstrates how genetic recombination creates novel morphology that has important implications in plant defenses. Loveland et al. investigated the impact of structural variants on gene expression. They tested whether genes located in chromosomal inversions creating three morphs of the ruff *Philomachus pugnax* differ in gene expression. They demonstrated a clear expression difference between morphs for CENPN, a gene disrupted by the inversion. Genes located inside the inverted genomic area, including genes coding for TFs and sex hormones, displayed tissue specific allelic imbalance, suggesting complex and wide-ranging changes in gene expression caused by structural variants.

Aviña-Padilla et al. inferred the conservation and functions of intronless genes (IGs), most of them originating from retrotransposition, in vertebrate genomes. Interestingly, many IGs code for TFs and other molecules involved in gene expression regulation. Comparative analysis confirmed high conservation of IGs, but revealed also a large proportion of evolutionary young IGs. These findings strengthen previous observations that IGs and multi-exon genes are under different evolutionary constraints.

Direct involvement of TFs in evolutionary processes was tested in two studies. The first, by Wang et al., used RNAseq-based analysis of genetic networks in high and low-altitude adapted pigs. A particular regulatory circuit triggered by a TF, KLF4, and involving TGF-β signaling active in the lungs of high-altitude pigs may have helped them to adapt to hypoxic environments. Jovanovic et al. investigated the evolution of more than 3000 gene regulatory factors (GRF) genes across 27 primate genomes and reported five candidate GRFs that have been positively selected on the human branch. Without finding common patterns of co-expression, the authors concluded that positively selected sites could have pleiotropic effects on phenotypic adaptation.

GRFs are one side of the coin, while their binding sites present the other. Mohanty explored the promoter architecture associated with transcriptional and hormonal regulation in diverse rice genotypes that provide varying tolerance to submergence. Analyzing transcriptome data, he identified putative *cis*-elements in promoters of genes upregulated in each tolerance group along with the TFs from each genotype. He surmised that phenotypic differences are due to the differences in transcriptional regulation across the groups. Evolution of *cis*-regulatory elements is also in the focus of the review from Joshi et al. In particular, they discuss studies on genetic variation in these elements within the context of population genetics. They introduce common approaches to identify regulatory regions and some statistical tools for inferring selection on non-coding functional regions. The authors highlight studies on the analyses of selective forces acting on non-coding genomic elements.

Finally, Weiner and Katz propose in their opinion paper that epigenetic processes are involved in generating the tremendous phenotypic diversity of protists by driving plasticity, differential adaptation and diversification. Their argument is based upon findings demonstrating that epigenetic processes are widespread across eukaryotes and that some epigenetic marks can be inherited.

Together, these articles represent a broad perspective on the role of gene regulation in speciation and adaptation. They cover a variety of scientific questions and approaches, both methodological and conceptual, over diverse taxonomic groups. Most importantly, they highlight that although an abundance of work on regulatory drivers of speciation and adaptation already exists, the ever-expanding amount of genomic data and techniques will continue to push for new questions and discoveries in this compelling field.

## References

[B1] Jones F. C. Grabherr M. G. Grabherr M. G. Chan Y. F. Russell P. Mauceli E. (2012). The Genomic Basis of Adaptive Evolution in Threespine Sticklebacks. Nature 484 (7392), 55–61. 10.1038/nature10944 22481358 PMC3322419

[B2] Mack K. L. Ballinger M. A. Phifer-Rixey M. Nachman M. W. (2018). Gene Regulation Underlies Environmental Adaptation in House Mice. Genome Res. 28 (11), 1636–1645. 10.1101/gr.238998.118 30194096 PMC6211637

[B3] Wolf J. B. W. Lindell J. Backström N. (2010). Speciation Genetics: Current Status and Evolving Approaches. Philos. Trans. R. Soc. B 365 (1547), 1717–1733. 10.1098/rstb.2010.0023 PMC2871893 20439277

